# Role of Dapagliflozin in Ischemic Preconditioning in Patients with Symptomatic Coronary Artery Disease—DAPA-IP Study Protocol

**DOI:** 10.3390/ph17070920

**Published:** 2024-07-10

**Authors:** Marco Alexander Valverde Akamine, Beatriz Moreira Ayub Ferreira Soares, João Paulo Mota Telles, Arthur Cicupira Rodrigues de Assis, Gabriela Nicole Valverde Rodriguez, Paulo Rogério Soares, William Azem Chalela, Thiago Luis Scudeler

**Affiliations:** Instituto do Coração (InCor), Hospital das Clínicas da Faculdade de Medicina da Universidade de São Paulo, Av. Dr. Enéas de Carvalho Aguiar, 44, Cerqueira César, São Paulo 05403-000, Brazil; marco.akamine@hc.usp.br (M.A.V.A.); bia.soares@incor.usp.br (B.M.A.F.S.); joao.telles@fm.usp.br (J.P.M.T.); arthurcicupira@usp.br (A.C.R.d.A.); gabriela.rodriguez@fm.usp.br (G.N.V.R.); paulo.soares@hc.fm.usp.br (P.R.S.); william.chalela@incor.usp.br (W.A.C.)

**Keywords:** ischemic preconditioning, SGLT2 inhibitors, myocardial ischemia, coronary artery disease

## Abstract

**Background:** Ischemic preconditioning (IP) is a powerful cellular protection mechanism. The cellular pathways underlying IP are extremely complex and involve the participation of cell triggers, intracellular signaling pathways, and end-effectors. Experimental studies have shown that sodium-glucose transport protein 2 (SGLT2) inhibitors promote activation of 5′-adenosine monophosphate (AMP)-activated protein kinase (AMPK), the main regulator of adenosine 5′-triphosphate homeostasis and energy metabolism in the body. Despite its cardioprotective profile demonstrated by numerous clinical trials, the results of studies on the action of SGLT2 inhibitors in IP are scarce. This study will investigate the effects of dapagliflozin on IP in patients with coronary artery disease (CAD). **Methods:** The study will include 50 patients with multivessel CAD, ischemia documented by stress testing, and preserved left ventricular ejection fraction (LVEF). Patients will undergo four exercise tests, the first two with a time interval of 30 min between them after washout of cardiovascular or hypoglycemic medications and the last two after 7 days of dapagliflozin 10 mg once a day, also with a time interval of 30 min between them. **Discussion:** The role of SGLT2 inhibitors on IP is not clearly established. Several clinical trials have shown that SGLT2 inhibitors reduce the occurrence cardiovascular events, notably heart failure. However, such studies have not shown beneficial metabolic effects of SGLT2 inhibitors, such as reducing myocardial infarction or stroke. On the other hand, experimental studies with animal models have shown the beneficial effects of SGLT2 inhibitors on IP, a mechanism that confers cardiac and vascular protection from subsequent ischemia–reperfusion (IR) injury. This is the first clinical study to evaluate the effects of SGLT2 inhibitors on IP, which could result in an important advance in the treatment of patients with stable CAD.

## 1. Background

Ischemic preconditioning (IP) is recognized as a protective cellular mechanism in which brief, recurrent episodes of myocardial ischemia followed by reperfusion can self-protect the heart from prolonged ischemic injury and thereby limit myocardial infarction size [1].

In humans, IP can be assessed in different scenarios, such as percutaneous coronary intervention (PCI), intermittent aortic clamping during coronary artery bypass graft surgery, and sequential stress tests [2,3,4]. The phenomenon of “warm-up” or “walk-through angina” has been related to IP and documented in several studies [5,6,7,8]. Improvement in ischemic parameters, such as the time to reach 1.0 mm ST segment deviation (ST) on electrocardiogram (ECG) and the time to develop angina in two sequential tests is considered a manifestation of IP.

The cellular pathways underlying IP are not fully understood and are undoubtedly complex, involving triggers, mediators, memory, and end-effectors. All steps appear to involve components such as adenosine, adenosine receptors, the activation of the adenosine triphosphate-sensitive potassium channels (K_ATP_ channels) and the Ɛ-isoform of protein kinase C, and others including the paradoxical protective role of oxygen radicals [9]. Opening of the K_ATP_ channel in cardiac myocytes has been consistently observed to be important in contributing to protection against IP [10].

IP typically results in increased gene expression and cellular metabolism [11]. A central target of such changes in gene expression and metabolism is the mitochondria. The direct and indirect effects of IP on mitochondria can result in the activation of adenosine monophosphate-activated protein kinase (AMPK), a regulator of cellular metabolism [11].

It has been established that anaerobic glycolysis is important for the generation of ATP during ischemia and crucial for the attenuation of ischemic injury in the heart [12,13]. Increased glucose uptake in ischemic cardiomyocytes is achieved mainly by translocation of a glucose transporter, GLUT4, from its intracellular compartments to the sarcolemma [14,15]. It is known that the expression level of GLUT4 is increased by AMPK activation [16].

Thus, as AMPK is a protein that plays an important role in regulating myocardial energy metabolism, reducing ischemia/reperfusion (I/R) injury; it may have beneficial effects on numerous cardiovascular diseases, such as heart failure and ventricular remodeling, vascular endothelial dysfunction, chronic inflammation, apoptosis, and regulation of autophagy [17].

An increased intracellular ratio of AMP to ATP, as occurs during strenuous exercise, hypoxia, or nutritional deficiency, can phosphorylate a threonine, the 172nd amino acid of the α subunit, thereby activating AMPK [18].

Upon activation, AMPK shuts down ATP consumption pathways and activates catabolic ATP production pathways through downstream signaling and target molecules [19,20], regulating lipid and protein metabolism, fatty acid oxidation, glucose uptake, gluconeogenesis, and autophagy [21,22]. AMPK also plays an important role in reducing oxidative stress by regulating autophagy and anti-apoptosis of cardiomyocytes [23,24].

Several oral hypoglycemic agents are capable of promoting the activation of AMPK, including sodium-glucose transport protein 2 (SGLT2) inhibitors. This class of medication selectively inhibits SGLT2 in the renal proximal tubule, with a consequent decrease in renal tubular thresholds for glycosuria and an increase in urinary glucose excretion, reducing blood glucose independently of insulin.

After clinical analysis, the protective effect of SGLT2 inhibitors on the heart may be related to reduced blood pressure, weight loss, decreased serum uric acid level, osmotic diuresis, reduced volume load, and hemodynamic changes [25].

Currently, basic research has focused on the action of this class on energy metabolism, inflammation, oxidative stress, myocardial fibrosis and electrolyte homeostasis [26]. SGLT2 inhibitors change the energy metabolism of the heart from glucose to fat [27,28,29] and slightly increase the ketone level [30], which is beneficial for energy supply to the heart. Dapagliflozin may delay the occurrence and progress of diabetic cardiomyopathy [31].

Few studies have evaluated the effects of SGLT2 inhibitors on AMPK. Canagliflozin activates AMPK of human embryonic kidney cells (HEK-293) and hepatocytes by inhibiting complex I in the mitochondrial respiratory chain and increasing cellular AMP levels [32]. Clinically relevant concentrations of canagliflozin can directly inhibit the secretion of endothelial pro-inflammatory chemokines/cytokines by AMPK-dependent and -independent mechanisms without affecting early interleukin-1β (IL-1β) signaling [33]. Zhou et al. showed in a murine model that empagliflozin is capable of reducing injury to diabetic cardiac microvascular endothelial cells (CMECs) by inhibiting mitochondrial fission through activation of the AMPK-Drp1 (dynamin-related protein 1) signaling pathways, can preserve cardiac barrier function of CMECs by suppressing mitochondrial reactive oxygen species production, and can subsequently reduce oxidative stress by inhibiting CMEC senescence [34].

In turn, dapagliflozin has been shown in animal models to be capable of decreasing the activation of the NOD-like receptor family, pyrin domain containing protein 3/apoptosis-associated speck-like containing a CARD inflammasome (NLRP3/ASC), thereby attenuating myocardial inflammation, fibrosis, apoptosis, and diabetic remodeling likely mediated through AMPK activation [35]. Additionally, Tsai et al. showed that dapagliflozin reduced the H/R-elicited oxidative stress via modulation of AMPK [36]. Recently, Tanajaket al. showed that dapagliflozin has greater cardioprotective efficacy than vildagliptin in rats with cardiac I/R injury [37].

Despite its cardioprotective profile demonstrated by numerous clinical trials [38,39,40], the results of studies on the action of SGLT2 inhibitors in IP are scarce. The few studies that have addressed this question have been carried out in animal models [37,41,42]. Furthermore, in humans, there are no studies that have evaluated the effects of dapagliflozin on IP expression. Therefore, the objective of the present study will be to evaluate the effects of dapagliflozin on IP in patients with stable, symptomatic, multivessel coronary artery disease (CAD) and preserved left ventricular ejection fraction (LVEF).

## 2. Methods/Design

A total of 50 patients with documented multivessel CAD, ischemia documented by stress testing, and preserved left ventricular ejection fraction (LVEF) will be screened (Table 1).

CAD will be documented by coronary angiography by visual assessment of atherosclerotic lesions with luminal obstruction of more than 70% in at least two different coronary territories.

Left ventricular systolic function will be measured by transthoracic echocardiography or ventriculography and will be considered preserved if the LVEF is greater than 50%.

Exercise treadmill tests (ETTs) that result in depression of the ST segment during effort greater than or equal to 1.0 mm, horizontal or descending and associated or not with angina, will be considered positive.

Exclusion criteria are shown in Table 2.

### 2.1. Patient Preparation

After clinical and cardiological evaluation, patients will be instructed to stop medications with cardiovascular effects that potentially interfere with IP before sequential exercise tests, depending on the half-life of the drug. Diabetic patients will be instructed to suspend, in addition to medications with cardiovascular effects, oral hypoglycemic agents for a similar period before the tests. Only nitrates will be maintained, when necessary, up to 24 h before testing.

Patients will be instructed not to perform physical activities during the period and to control their salt intake, and patients with diabetes will be advised to strictly control their carbohydrate intake. They will also be instructed to contact the study team by telephone, who will be available 24 h a day, in case of questions or worsening of symptoms. On the day of the exams, the symptoms will be reassessed by the medical team before carrying out the sequential exercise tests.

### 2.2. Sequential Ergometric Tests

**Figure 1:**
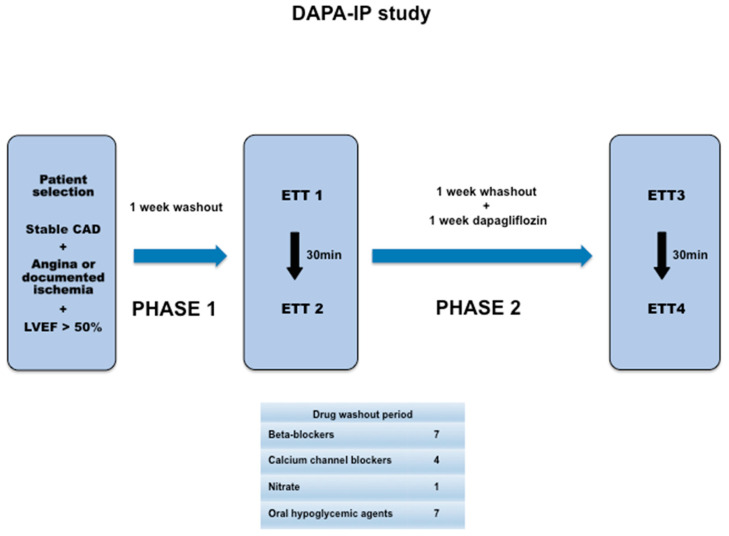
Study design. Legend: CAD: coronary artery disease; ETT: exercise treadmill test; LVEF: left ventricular ejection fraction.

The study protocol will include 2 phases (Figure 1). The criterion used to evaluate the IP took into account the methodology used in previous studies [43,44]. In Phase 1, after the washout period of cardiovascular or hypoglycemic medications, all patients will undergo 2 consecutive ETT (ETT1 and ETT2), with a 30 min interval between them to identify the ischemia and document the magnitude of IP by the difference in ischemia parameters between the 2 tests. The protocol will be adopted according to the assessment of each patient’s functionality (Bruce or modified Bruce). The ergometer used will be the GE T2100 Ergometric Treadmill coupled with a GE Case V6.73 system/software and a Tango M2 blood pressure monitor.

The recording system used will be 12 leads, including the classic leads of the Mason and Likar systems. Electrocardiographic recordings will be carried out in a standardized way, pre-exertion, every 5 to 10 s at a time close to T-1.0 mm, at the peak of exercise, at the time of the worst electrocardiographic change, at the time of arrhythmias, and at every minute of the recovery, which will last for 6 min.

Exercise tests that result in depression of the ST segment during effort greater than or equal to 1.0 mm, horizontal or descending, associated or not with chest pain will be considered positive.

Heart rate will be continuously monitored and documented every 15 s. Blood pressure measurement will be performed every 90 s, at the moment of T-1.0 mm, at peak effort and every minute of the recovery phase. The double product or rate pressure product (RPP) will be calculated by multiplying the heart rate in beats per minute (bpm) by the blood pressure in millimeters of mercury (mmHg), this variable being measured at the time of T-1.0 mm.

The criteria for interrupting exams will be those adopted by the recommendations of the Brazilian Society of Cardiology Guidelines [43].

After Phase 1, all patients will receive dapagliflozin at a dose of 10 mg once a day for 6 days. On the seventh day, patients will receive dapagliflozin 10 mg and will again undergo 2 consecutive ETTs (ETT3 and ETT4) 2 h after medication administration (time to reach peak plasma concentration) [Phase 2]. The time interval between ETT3 and ETT4 will be similar to that of Phase 1, that is, 30 min.

### 2.3. Characterization of IP

During sequential ETTs, confirmation of T-1.0 mm will be carried out by two experienced cardiologists, independently and blind to the sequence of tests. Situations of disagreement will be resolved by consensus. An improvement equal to or greater than 30 s in the time to reach depression in 1.0 mm of the ST segment in the second sequential test compared to T-1.0 mm in the first test will be classified as IP present. The second criterion adopted to characterize the IP will be used based on the analysis of the RPP result. Therefore, when the improvement of T-1.0 mm is borderline, the RPP at the time of T-1.0 mm will be considered. If this is higher in the second test compared to the first, the IP will be considered present.

### 2.4. Statistical Analysis

A two-way ANOVA with repeated measures followed by the Bonferroni test will be used to compare T-1.0 mm and RPP data. Comparisons of the remaining continuous or discrete variables between the two phases will be performed using an unpaired Student’s *t*-test or χ^2^, respectively. Fisher’s test will be used when appropriate. The data will be expressed as mean standard deviation and in the figures as median and interquartile ranges. A value of *p* < 0.05 will be considered significant. SPSS version 20 software will be used for all statistical analyses.

### 2.5. Sample Size Calculation

The primary outcome of the study will be the difference in the time required to achieve 1.0 mm of ST segment depression (T-1.0 mm) between the ETT3 and ETT1 [ETT3-ETT1] and ETT4 and ETT2 [ETT4-ETT2]. A sample of 50 patients will be necessary in order to detect a 33% increase in the time to reach T-1.0 mm with dapagliflozin compared to not using this medication, with a power of 80% and a two-sided significance level of 0.05 [45].

## 3. Discussion

Myocardial IP is an intracellular protective mechanism in which short periods of ischemia followed by reperfusion trigger cell signaling processes and cascades, which culminate in greater tissue resistance to subsequent ischemic insult.

The role of SGLT2 inhibitors on IP is not clearly established. Several clinical trials have shown that SGLT2 inhibitors reduce cardiovascular events, notably heart failure. However, such studies have not shown beneficial metabolic effects of SGLT2 inhibitors, such as reducing myocardial infarction or stroke. On the other hand, experimental studies with animal models have shown beneficial effects of SGLT2 inhibitors on IP, a mechanism that confers cardiac and vascular protection from subsequent ischemia–reperfusion (IR) injury. A recent meta-analysis of 16 independent animal models experiments that compared SGLT2i to a control showed that independent of diabetes, SGLT2is were significantly associated with fewer myocardial ischemia–reperfusion injuries and smaller infarct size [46]. The probable cellular mechanism that justifies the effects of dapagliflozin on cardiac ischemia/reperfusion (I/R) injury is through the modulation of AMPK. 

## 4. Results

The trial is ongoing. Recruitment for the main trial commenced on 15 December 2022. The expected recruitment completion date is July 2025.

## 5. Conclusions

The DAPA-IP trial will evaluate the effects of dapagliflozin on IP and may establish a new treatment option for stable CAD patients.

## Figures and Tables

**Table 1:** Inclusion criteria.

- Stable multivessel CAD (obstruction greater than 70% in at least two main coronary branches).
- LVEF ≥ 0.50, confirmed by transthoracic Doppler echocardiography.
- Documentation of stress-induced myocardial ischemia (horizontal or descending ST segment depression ≥1.0 mm)

**Table 2:** Exclusion criteria.

- Kidney failure (creatinine clearance < 60 mL/min)
- Severe liver failure
- Single-vessel CAD
- Myocardial infarction in the last 3 months
- LVEF < 50%
- Presence of any non-ischemic cardiomyopathy
- Moderate or severe valve disease
- Morphological changes in the QRS of the ECG and conduction defects that may interfere with the interpretation of changes in the ST segment
- Recent and negative exercise test for myocardial ischemia
- Positive exercise test for myocardial ischemia, with signs of high risk
- Limiting anginal symptoms or recent worsening
- Arrhythmias that make it difficult to characterize myocardial ischemia during exercise stress (atrial fibrillation or flutter)
- Patient refusal to participate in the study

## Data Availability

The data in this study are available from the corresponding author on reasonable request.
